# On the Modeling of the Donor/Acceptor Compensation Ratio in Carbon-Doped GaN to Univocally Reproduce Breakdown Voltage and Current Collapse in Lateral GaN Power HEMTs

**DOI:** 10.3390/mi12060709

**Published:** 2021-06-16

**Authors:** Nicolò Zagni, Alessandro Chini, Francesco Maria Puglisi, Paolo Pavan, Giovanni Verzellesi

**Affiliations:** 1 Department of Engineering “Enzo Ferrari”, University of Modena and Reggio Emilia, via P. Vivarelli 10, 41125 Modena, Italy; alessandro.chini@unimore.it (A.C.); francescomaria.puglisi@unimore.it (F.M.P.); paolo.pavan@unimore.it (P.P.); 2 Department of Sciences and Methods for Engineering (DISMI) and EN&TECH Center, University of Modena and Reggio Emilia, via G. Amendola, 2, 42122 Reggio Emilia, Italy; giovanni.verzellesi@unimore.it

**Keywords:** GaN power HEMTs, breakdown voltage, current collapse, compensation ratio, auto-compensation, carbon doping

## Abstract

The intentional doping of lateral GaN power high electron mobility transistors (HEMTs) with carbon (C) impurities is a common technique to reduce buffer conductivity and increase breakdown voltage. Due to the introduction of trap levels in the GaN bandgap, it is well known that these impurities give rise to dispersion, leading to the so-called “current collapse” as a collateral effect. Moreover, first-principles calculations and experimental evidence point out that C introduces trap levels of both acceptor and donor types. Here, we report on the modeling of the donor/acceptor compensation ratio (CR), that is, the ratio between the density of donors and acceptors associated with C doping, to consistently and univocally reproduce experimental breakdown voltage (*V*_BD_) and current-collapse magnitude (Δ*I*_CC_). By means of calibrated numerical device simulations, we confirm that Δ*I*_CC_ is controlled by the effective trap concentration (i.e., the difference between the acceptor and donor densities), but we show that it is the total trap concentration (i.e., the sum of acceptor and donor densities) that determines *V*_BD_, such that a significant CR of at least 50% (depending on the technology) must be assumed to explain both phenomena quantitatively. The results presented in this work contribute to clarifying several previous reports, and are helpful to device engineers interested in modeling C-doped lateral GaN power HEMTs.

## 1. Introduction

Carbon (C) doping is a common technological solution to reduce buffer conductivity and increase breakdown voltage (*V*_BD_) in lateral gallium nitride (GaN)-based power transistors [1,2]. However, this comes at the cost of increased dynamic on-resistance and current-collapse effects [1,3,4]. Depending on the growth conditions, C atoms can either substitute N or Ga sites, occupy interstitial locations in the crystal, or form complexes with intrinsic defects [5,6,7,8]. In typical undoped GaN layers used as buffer in high electron mobility transistors (HEMTs), the position of the Fermi level is such that both acceptor and donor traps are likely to form. Several works, discussing either simulation or experimental results, indicate the occurrence of partial “auto-compensation” between the dominant deep acceptor traps, generally attributed to C_N_ levels, and the concomitantly introduced (i.e., non-pre-existing) shallow donors, which reduce the *effective* concentration of acceptor traps well below the level of the introduced C concentration (especially in the case of extrinsic C doping) [4,9,10,11,12,13,14,15].

These aspects call for the correct modeling of C-related trap states in GaN transistors when performing device simulations to investigate important performance-limiting effects, such as buffer leakage and related *V*_BD_ [13,14], dynamic *R*_ON_ [4,10,16], current collapse [2,12,17], and threshold voltage instabilities [18,19,20,21]. In fact, both the concentration of acceptor states (*N*_C,A_) and donor states (*N*_C,D_), as well as their *compensation ratio*, defined as CR = *N*_C,D_/*N*_C,A_, need to be properly determined in order to reproduce the features of realistic devices and calibrate device simulation for a given technology.

In this paper, we present calibrated numerical device simulation results that reveal the functional dependence of *V*_BD_ and Δ*I*_CC_, respectively, on the *total* (*N*_C,TOT_ = *N*_C,A_ + *N*_C,D_) and *effective* (*N*_C,EFF_ = *N*_C,A_ − *N*_C,D_) C-related trap concentrations in the buffer at different CR values. Our results (i) confirm the necessity of assuming compensating donor traps in C-doped GaN to correctly model realistic devices, and (ii) provide physical insights into the origin of the observed *V*_BD_ and Δ*I*_CC_ dependence on CR.

## 2. Modeling Framework

**Figure 1:**
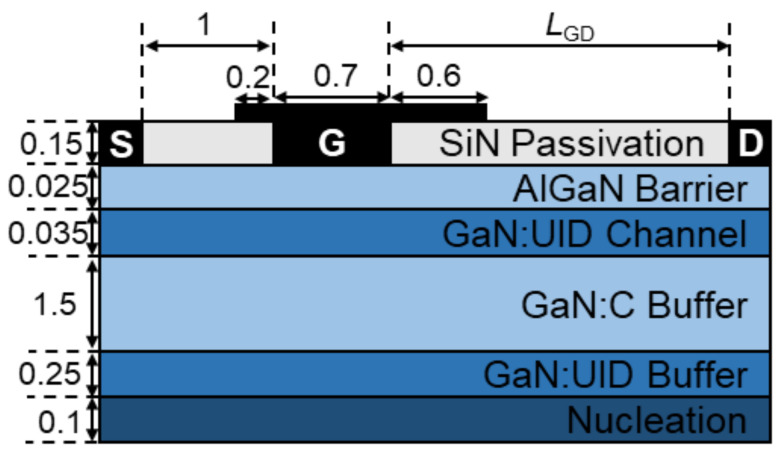
Cross-section of the simulated Schottky-gate HEMT resembling the C-doped device in [1]. Dimensions are in μm (not to scale).

Two-dimensional numerical device simulations were carried out with the commercial simulator SDevice^TM^. The simulated structure is sketched in Figure 1 with indication of device dimensions (not to scale); the device resembles the AlGaN/GaN Schottky-gate HEMT reported in [1].

Charge transport was modelled by means of the drift-diffusion model. Piezoelectric polarization was included by using the default strain model of the simulator. Note that at the passivation/barrier interface, the polarization model was deactivated. This approach is equivalent to assuming that the negative polarization charge at this interface is completely compensated by an equal positive surface charge [22]. Therefore, we neglected the possible dynamic effects related to surface traps.

Chynoweth’s law was used to model impact ionization; model coefficients for both electrons and holes were set in agreement with Monte Carlo calculations [23].

Gate current was modelled by the thermionic and field emission mechanisms. The field emission component was calculated self-consistently by the simulator through a nonlocal tunnelling model based on the WKB approximation [16].

To account for trap effects, one Shockley-Read–Hall (SRH) trap-balance equation was used for each distinct trap allowing for the dynamics of trap occupation to be described without any quasi-static approximation.

Calibration of the simulation parameters against measurements taken from [1] has already been reported in [14,17]. What makes the measurements reported in [1] instrumental to our scope is the possibility of calibrating our simulation deck against a consistent set of experimental data from devices with several different *L*_GD_ values.

**Figure 2:**
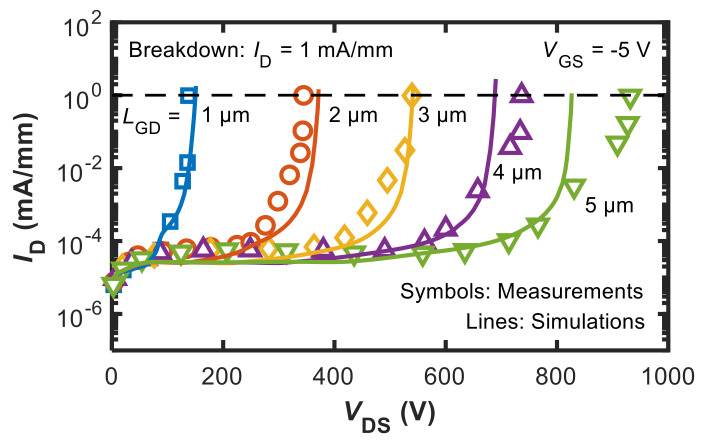
Calibrated simulations (lines) and measurements (symbols) of the off-state *I*_D_–*V*_DS_ curves. Measured data are taken from [1]. Adapted from [14].

**Figure 3:**
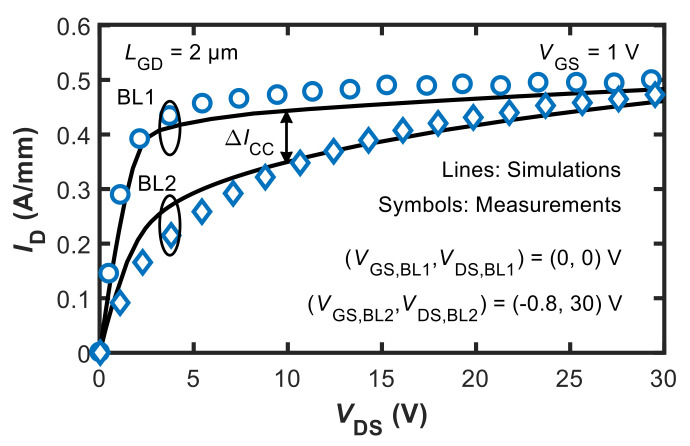
Calibrated pulsed *I*_D_–*V*_DS_ curve simulations (lines) showing the achieved agreement in current-collapse measurements from [1] (symbols). Adapted from [17].

Key results are shown in Figure 2 and Figure 3, illustrating the agreement achieved in the off-state three-terminal breakdown and current collapse, respectively. Regarding the pulsed *I*_D_–*V*_DS_ curves shown in Figure 3, the output curves were obtained by pulsing *V*_GS_ and *V*_DS_ from different baselines to either suppress or induce trapping [1]. The current collapse is defined as Δ*I*_CC_ = (*I*_D,BL1_ − *I*_D,BL2_)/*I*_D,BL1_ × 100 evaluated at *V*_DS_ = 10 V.

C doping in the GaN buffer was modelled by considering a dominant deep acceptor trap at *E*_V_ + 0.9 eV (generally assumed to correspond to C_N_ states) and a shallow donor trap at *E*_C_ − 0.11 eV (more likely related to C_Ga_ states) as the two major energy states associated with C [24]. When varying trap concentrations and CR, the above energy levels were kept fixed and other possible states related to C doping were neglected. The adopted concentrations for the calibrations shown in Figure 2 and Figure 3 were 8 × 10^17^ cm^−3^ and 4 × 10^17^ cm^−3^ for C-related acceptors and donors, respectively.

Although no additional trap levels were considered, in all nitride layers a background doping concentration of 10^15^ cm^−3^ was adopted to account for the unintentional n-type conductivity due to shallow-donor impurities incorporated during growth [2,13]. The C doping model based on discrete point defects adopted here can lose validity for impurity concentrations larger than 10^19^ cm^−3^, for which a dominant defect band behavior has been proposed to be more appropriate [11]. Therefore, we limited our analysis to cases for which *N*_C,A_ < 10^19^ cm^−3^.

As elucidated by the results in Section 3, the key feature of the adopted C doping model is that the dominant deep acceptor-type hole traps are partially compensated by shallow donor-type electron traps. Note that the actual energy position of donor traps, if sufficiently shallow, has little influence on the simulation results. C-related donors could actually be moved even closer to *E*_C_ or be modelled as completely ionized dopants (i.e., fixed positive charge) [25], in agreement with recent hybrid-functional density functional theory (DFT) calculations [6,8], without significant changes. This is because as long as the dominant traps are the acceptor states at *E*_V_ + 0.9 eV, the Fermi level stays well below the shallow energy of donors, thus guaranteeing their complete ionization. The capability of the acceptor–donor model for C doping to reproduce source–drain leakage currents and off-state breakdown is shown in [13,14].

Table 1 lists the main physical mechanisms, along with the respective models and parameters included in the simulations.

## 3. Results

To understand the impact of the total (*N*_C,TOT_ = *N*_C,A_ + *N*_C,D_) and effective (*N*_C,EFF_ = *N*_C,A_ − *N*_C,D_) C-related trap concentration on *V*_BD_ and Δ*I*_CC_, we performed a sensitivity analysis starting from the parameter set, resulting in the calibrated results shown in Figure 2 and Figure 3. *L*_GD_ was set to 2 μm because only for this case, both *V*_BD_ and Δ*I*_CC_ measurement data were available in [1]. Three different CR values were considered for simplicity in the following: 0%, 50%, and 90%. For each CR value, *N*_C,A_ was set to {0.04, 0.08, 0.4, 0.8, 4, 8, 40, 80} × 10^17^ cm^−3^, while *N*_C,D_ was set according to the assumed CR (i.e., 0%, 50%, or 90% of *N*_C,A_).

### 3.1. Breakdown Voltage

**Figure 4:**
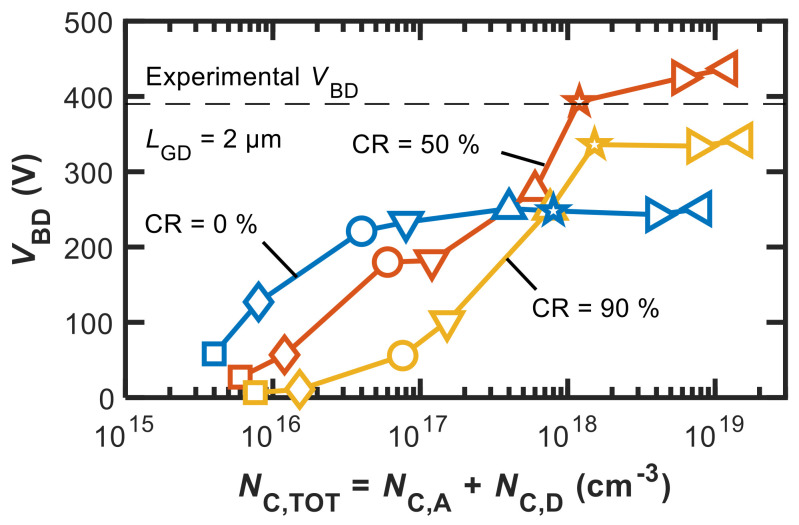
*V*_BD_ vs. *N*_C,TOT_ for CR = {0, 50, 90}%. The same symbols correspond to the same *N*_C,A_ at different *N*_C,D_ depending on CR.

Figure 4 shows *V*_BD_ as a function of *N*_C,TOT_ for the different CR values. As can be noted, for all CR values, *V*_BD_ first increases and then saturates with *N*_C,TOT_.

This behaviour is largely expected, as it is related to the decrease in the electric field peak at the gate edge resulting from the increase in the ionized acceptor density (negative charge). In essence, it is exactly to induce this effect that doping with acceptor impurities (like Fe and C) is adopted in power GaN HEMTs. The *V*_BD_ saturation is attributable to the fact that once the electric-field peak moves from the gate edge to the drain contact, the beneficial effect of further increasing the acceptor concentration ceases.

**Figure 5:**
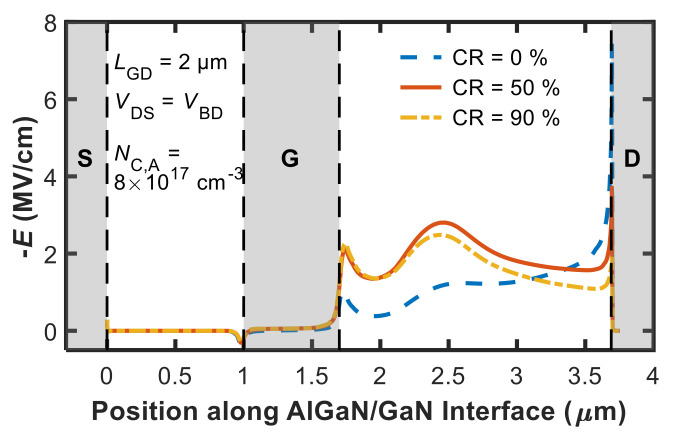
Lateral component of the electric field (*E*) along the AlGaN/GaN interface at breakdown (*V*_GS_ = −5 V, *V*_DS_ = *V*_BD_) for CR = {0, 50, 90}% at the same *N*_C,A_ value (8 × 10^17^ cm^−3^).

**Figure 6:**
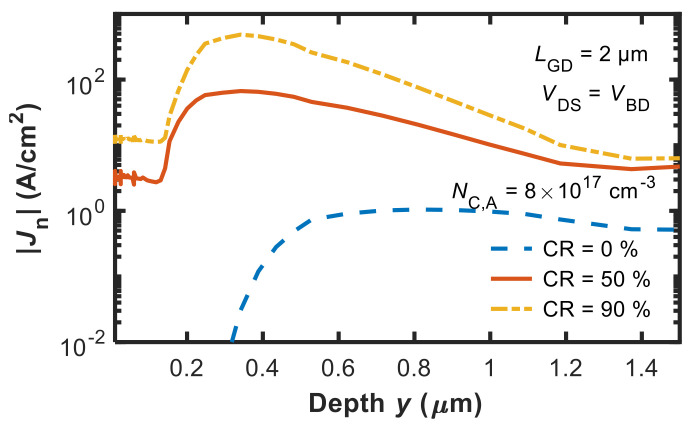
Modulus of the electron current density (|*J*_N_|) as a function of the device depth along a cutline taken in the middle of the gate contact for CR = {0, 50, 90}% at the same *N*_C,A_ value (8 × 10^17^ cm^−3^).

In addition to confirming the above behaviour, Figure 4 provides us with two other pieces of information that are key to our purposes: (1) the maximum *V*_BD_ attainable at large *N*_C,TOT_ (*V*_BD,max_) is a non-monotonic function of CR, and (2) a significant CR value of about 50% must be assumed in order to obtain *V*_BD_ in agreement with experiments. The reasons behind this can be understood with the aid of Figure 5 and Figure 6.

Figure 5 shows the lateral component of the electric field as a function of position along a cutline corresponding to the AlGaN/GaN interface at breakdown (*V*_GS_ = −5 V, *V*_DS_ = *V*_BD_) for *N*_C,A_ = 8 × 10^17^ cm^−3^ in the three cases of CR = 0%, 50%, and 90%. As shown in Figure 5, increasing CR from 0% (i.e., *N*_C,A_ = 8 × 10^17^ cm^−3^, *N*_D,A_ = 0 cm^−3^) to 50% (i.e., *N*_C,A_ = 8 × 10^17^ cm^−3^, *N*_D,A_ = 4 × 10^17^ cm^−3^) and 90% (i.e., *N*_C,A_ = 8 × 10^17^ cm^−3^, *N*_D,A_ = 7.2 × 10^17^ cm^−3^) effectively modulates the electric field profile, relaxing the peak at the drain contact. Positively charged donors thus contribute to make the electric field profile more uniform (if the electric field peak has already moved to the drain contact). This explains why in Figure 4, *V*_BD,max_ increases from about 250 V to about 450 V as CR is raised from 0% to 50%.

When further increasing CR to 90%, *V*_BD,max_ is reduced. This behavior can be explained with the aid of Figure 6, which shows the modulus of the electron current density as a function of the device depth along a vertical cutline taken in the middle of the gate contact. As is clearly shown, the source–drain punch-through (or sub-threshold) current across the buffer increases as CR increases from 0% to 50% and 90%. This is a consequence of the higher *N*_C,D_ that increases the conductivity of the buffer and thus reduces *V*_BD_.

In summary, without significant donor/acceptor compensation resulting in a CR of about 50%, it is not possible, according to our analysis, to explain the state-of-the-art *V*_BD_ vs. *L*_GD_ dependence with slopes of 150–200 V/mm [1,26,27,28], and, specifically in the case considered here, a *V*_BD_ of about 370 V for a device with an *L*_GD_ of 2 μm.

### 3.2. Current Collapse

**Figure 7:**
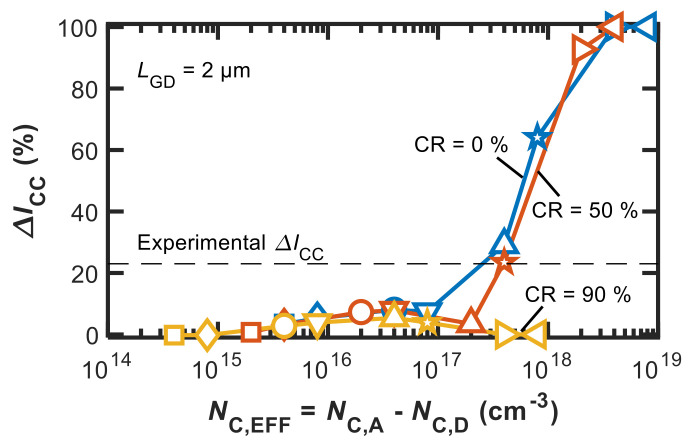
Δ*I*_D,CC_ vs. *N*_C,EFF_ for CR = {0, 50, 90}%. The same symbols correspond to the same *N*_C,A_ at different *N*_C,D_ depending on CR.

The results of the sensitivity analysis on Δ*I*_CC_ are shown in Figure 7, where Δ*I*_CC_ is plotted against *N*_C,EFF_ for the same CR values used for Figure 4. As it can be noted, Δ*I*_CC_ remains small (<10% in the specific devices considered here) regardless of CR when *N*_C,EFF_ is smaller than 10^17^ cm^−3^. For higher *N*_C,EFF_, unless CR is very large (90% in our case), Δ*I*_CC_ increases steeply with *N*_C,EFF_, reaching values >60%, which are well above those reported for state-of-the-art C-doped GaN power HEMTs for *N*_C,EFF_ values >10^18^ cm^−3^, the latter being instead quite typical for nominal C densities in extrinsically doped devices (i.e., using C precursors). In other words, according to this analysis it is unreasonable that C doping at high concentrations could simply translate to C_N_ acceptors, as in this case DC-to-dynamic dispersion effects as current collapse and dynamic *R*_ON_ increase would make the device completely nonfunctional.

This is in agreement with previous results that showed how assuming CR = 0% (i.e., acceptors only) with concentrations on the order of the nominal C density (i.e., ~10^18^–10^19^ cm^−3^) resulted in large overestimation of current-collapse effects measured in actual devices of different technologies [4,12,18,29].

## 4. Discussion

By combining the results shown in Figure 4 and Figure 7, we observe that the *V*_BD_ and Δ*I*_CC_ values measured in the device under study can be reproduced with a single set of parameters, and specifically, with the same *N*_C,TOT_ and *N*_C,EFF_, only when considering a CR of about 50%. More generally, our results point to the necessity that a non-negligible part of incorporated C atoms results in donor-like levels or contribute to donor-like defect-impurity centers, thus compensating to a significant degree the dominant acceptor traps introduced by C doping.

The results presented in this work are relevant for the modeling of any GaN HEMT structure that incorporates C impurities (even unintentionally) in significant concentrations. High unintentional C doping concentrations can likely occur for metal-organic chemical vapor deposition (MOCVD)-grown, intentionally Fe-doped HEMTs for RF applications, where C incorporation comes as an inevitable consequence of the growth processing conditions [30].

## 5. Conclusions

We reported on the modeling of the compensation ratio (CR) between the donor and acceptor densities due to carbon doping in the buffer of lateral GaN power HEMTs to correctly simulate breakdown voltage (*V*_BD_) and current collapse (Δ*I*_CC_). We showed that compensating shallow donor traps (*N*_C,D_) need to be considered in addition to the dominant deep acceptor traps (*N*_C,A_), in order to reproduce *V*_BD_ and Δ*I*_CC_ with a single set of parameters. Furthermore, we identified that the primary dependence of *V*_BD_ (Δ*I*_CC_) on C doping is through the total (effective) concentration of acceptor and donor traps. The results presented here allow device engineers to properly model a given GaN HEMT technology that incorporates C in its structure (even unintentionally) by setting the CR value required to univocally reproduce both *V*_BD_ and Δ*I*_CC_ data.

## Figures and Tables

**Table 1:** List of main physical mechanisms, the respective models, and parameters used in the simulations.

Physical Mechanism	Model	Parameters	Value
Impact Ionization	Chynoweth’s Law	*a* (electrons)	2.32 × 10^6^ cm^−1^
		*b* (electrons)	1.4 × 10^7^ V/cm
		*a* (holes)	5.41 × 10^6^ cm^−1^
		*b* (holes)	1.89 × 10^7^ V/cm
Carbon Doping (Buffer)	Acceptor Trap Level	Concentration	Variable
		Energy Level	0.9 + *E*_V_ eV
	Donor Trap Level	Concentration	Variable
		Energy Level	*E*_C_—0.11 eV
Unintentional Doping (Channel)	Donor Trap Level	Concentration	1 × 10^15^ cm^−3^
Schottky Diode (Gate Contact)	Thermionic and Field Emission	Schottky Barrier Height	1 eV
Low-Field Mobility (GaN)		*µ*_n_	1800 cm^2^/Vs
High-Field Saturation (GaN)	Canali Model	*v*_n,sat_	1.5 × 10^7^ cm/s
